# Reentrant Processing in Intuitive Perception

**DOI:** 10.1371/journal.pone.0009523

**Published:** 2010-03-04

**Authors:** Phan Luu, Alexandra Geyer, Cali Fidopiastis, Gwendolyn Campbell, Tracey Wheeler, Joseph Cohn, Don M. Tucker

**Affiliations:** 1 Electrical Geodesics, Inc., Eugene, Oregon, United States of America; 2 Aptima, Inc., Woburn, Massachusetts, United States of America; 3 Institute for Simulation and Training, University of Central Florida, Orlando, Florida, United States of America; 4 Naval Air Warfare Center Training Systems Division (NAWCTSD), Orlando, Florida, United States of America; 5 System Planning Corporation, Arlington, Virginia, United States of America; 6 Defense Sciences Office, Defense Advance Research Projects Agency, Arlington, Virginia, United States of America; Cuban Neuroscience Center, Cuba

## Abstract

The process of perception requires not only the brain's receipt of sensory data but also the meaningful organization of that data in relation to the perceptual experience held in memory. Although it typically results in a conscious percept, the process of perception is not fully conscious. Research on the neural substrates of human visual perception has suggested that regions of limbic cortex, including the medial orbital frontal cortex (mOFC), may contribute to intuitive judgments about perceptual events, such as guessing whether an object might be present in a briefly presented fragmented drawing. Examining dense array measures of cortical electrical activity during a modified Waterloo Gestalt Closure Task, results show, as expected, that activity in medial orbital frontal electrical responses (about 250 ms) was associated with intuitive judgments. Activity in the right temporal-parietal-occipital (TPO) region was found to predict mOFC (∼150 ms) activity and, in turn, was subsequently influenced by the mOFC at a later time (∼300 ms). The initial perception of gist or meaning of a visual stimulus in limbic networks may thus yield reentrant input to the visual areas to influence continued development of the percept. Before perception is completed, the initial representation of gist may support intuitive judgments about the ongoing perceptual process.

## Introduction

A common view is that perception begins with input to sensory cortex, and then continues with processing in visual association cortex to achieve the interpretation required for full perception. However, psychological studies of perception have suggested that memory operates early in the perceptual process, to bring both current expectancies and previous perceptual experience to the organization of sensory data into meaningful percepts [1], [2], [3]. Given the brain's reentrant (i.e., back projecting) connectional architecture that links each sensory modality with unimodal association, heteromodal association, and finally limbic cortex [4], a reasonable hypothesis is that the process of perception requires the reentrant corticolimbic mechanisms of memory consolidation, linking the multiple networks of the corticolimbic hierarchy.

When the perceptual process is incomplete, such as with limited sensory information, some access to the process appears to allow the person to draw limited inferences, often described as the “feeling of knowing” [5]. Considering the multiple networks linked in the perceptual process, we can infer that some limited conscious access to the initial response in limbic networks is important to the feeling of knowing, and thus to the intuitive monitoring of the perceptual process. Because limbic networks organize the visceral, evaluative base of cognition [6], [7], we can understand why the feeling of knowing is often affectively charged.

**Figure 1:**
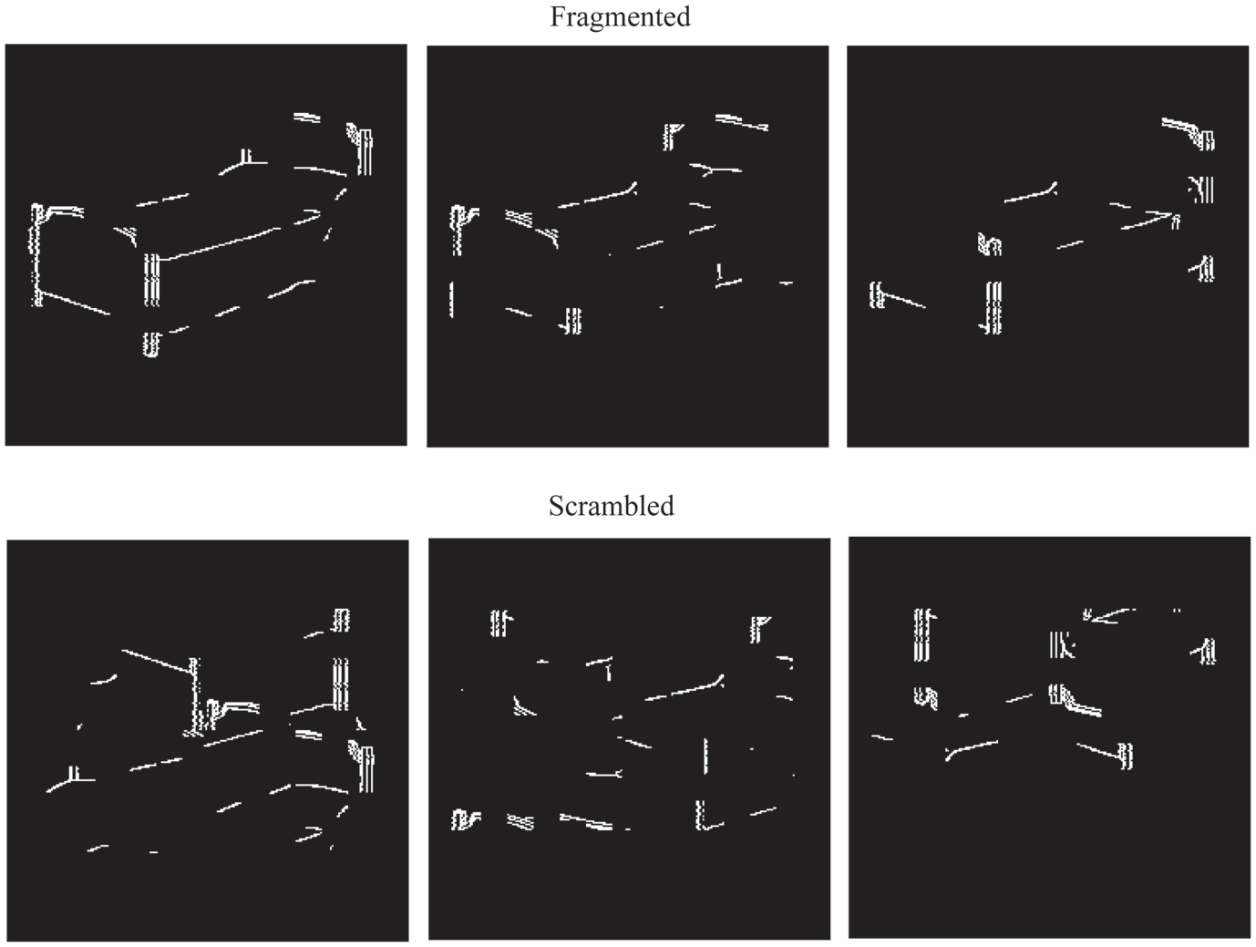
Sample stimuli. Top row: fragmented images (object is a bed) at three fragmentation levels. Bottom row: scrambled images of the object shown in the top row.

In a functional magnetic resonance imaging (fMRI) study, Volz and von Cramon [8] provided evidence of the limbic contribution to the intuitive process associated with visual perception. As they examined briefly presented fragmented or scrambled line drawings (see Figure 1), the participants in this experiment were asked to report when they perceived an object (i.e., coherence), even if they were not sure what it was. When a possible object was reported, there was increased hemodynamic response in the medial orbital frontal cortex (mOFC) compared to when participants reported not perceiving a possible object (i.e., no coherence). The mOFC is a region at the limbic base of the ventral frontal lobe that is closely interconnected with the insula, anterior temporal lobes, and other ventral limbic networks [4]. Volz and von Cramon interpreted their finding as consistent with other evidence showing mOFC activity when memory representations were important in guiding visual perception [9].

The notion of a limbic influence shaping the perceptual process seems to imply that the process is a developmental one, in which the meaning of the perception is not apprehended simply, but must be organized over time (even if this time is a few tens of milliseconds). Early psychological theories of memory-based stages in perception emphasized developmental or microgenetic nature of the perceptual process. There is a progressive articulation of the features of the percept over time, beginning with a global apprehension of the object and/or context, and then differentiating the elements and structure of the veridical percept [10]. More modern cognitive approaches to perception also emphasized the importance of global precedence, in which holistic features of the stimulus array are typically registered first, and more detailed local analysis often occurs later [11], [12], [3]. A neurological formulation of the microgenetic model aligned each stage of perceptual development with levels of the neuraxis, with the global apprehension of significance formed in the limbic base of sensory modalities, and only the final articulation of the conscious percept becoming fully constrained in the primary sensory cortices [13].

If it is true that the mechanisms of memory consolidation are responsible for the organizational process in perceptual development, then the architecture of the linked corticolimbic networks suggests the consolidation process is not linear, but reentrant. The “forward” projections, from primary sensory toward unimodal, then heteromodal association, and then limbic cortex, are matched at each level by equally numerous “back” projections, which appear to shape the sensory processing by constraints of the deeper networks, closer to the limbic hemispheric core. Considering the Volz and von Cramon [8] findings, if the response in mOFC signals that an object may be present, this response would become effective only as it feeds back to stimulate visual cortical networks to engage further perceptual processing [3].

Using the same visual task (i.e., the Modified Waterloo Gestalt Closure Task) employed by Volz and von Cramon [8], we examined reentrant processing in corticolimbic networks in the perceptual process where rapid, holistic processing would lead to intuitive-like judgments. Whereas the temporal resolution of the hemodynamic response assessed by fMRI is inadequate to separate fine distinctions in the sequence of neural processing, EEG measures provide millisecond temporal resolution that can resolve the temporal order of neural activity. To provide sufficient spatial resolution to the EEG measures, we applied a dense array EEG (dEEG) technology, with 256 recording channels distributed over the head surface allowing source localization of the electrical responses to at least sublobar accuracy [14]. Considering regions of interest defined in part by Volz and von Cramon's fMRI findings, we hypothesized, first, that the electrical response in mOFC would be greater for the judgment of object present than object absent, and, second, that the enhanced mOFC response would predict enhancement of a later stage of continued (reentrant) processing in visual networks.

## Methods

### Participants

Participants were recruited from the general student population at the University of Oregon. Twenty-two, right-handed participants completed the study (14 males). Participants' ranged between 18–43 years of age (mean  = 22, SD = 5.02). All participants had normal or corrected-to-normal vision. Participants reported no history of seizures or head injuries resulting in loss of consciousness, nor taking medications (e.g., anticonvulsants) or illicit drugs that could affect the EEG. Informed written consent was obtained from each participant prior to participation in the studies. The protocol was approved by EGI, the University of Oregon, and the University of Central Florida's institutional review boards.

### Stimuli

Images were taken from the Snodgrass and Vanderwart [15] inventory. The 4.5″ X 4.5″ images were then fragmented using the program Ultrafrag (Life Science Associates, Bayport, NY). Ultrafrag uses the method described in Snodgrass, Smith, Feenan, and Corwin [16] for removing blocks of pixels from an image. Three different levels of fragmentation were utilized (see Figure 1). To create the scrambled images, each image was divided into eight parts and these parts were randomly rearranged. Each part contained local collinearity but the overall image did not portray a coherent object. The scrambled images contained the identical number of pixels as the fragmented images.

### Tasks

A total of 200 unique images were used: 150 fragmented and 50 scrambled. Participants were never presented with an image containing the same object at different levels of fragmentation. Each trial began with a cue (an asterisk) lasting 500 ms. The cue alerted participants to the start of a trial, and it was replaced by a fixation cross lasting 500 ms. Following fixation cross, an image was presented for 400 ms followed by a fixation cross. The fixation cross was presented for two seconds. The response interval was from onset of an image to offset of the fixation cross (2.4 seconds). Inter-trial interval varied randomly between 1.5 and 2.5 seconds.

Participants were instructed to indicate whether an image contained a possible object. It was emphasized that participants should rely on impressions of whether or not the image was of a possible object and that they did not have to be able to name or identify the object in order to decide whether a possible object was present. To emphasize that accuracy was not the goal, no performance feedback was provided at the end of each trial. Trials were blocked into two blocks of 100 trials (75 fragmented, 25 scrambled). Within each block, fragmented and scrambled pictures were randomly presented.

### EEG Recording

The EEG was acquired using a 256-channel HydroCel Geodesic Sensor Net (EGI, Eugene, OR). All electrodes impedances were kept below 70 KΩ [17]. Recordings were referenced to Cz. The EEG was bandpass filtered (0.1–100 Hz) prior to being sampled at 250s/s with a 24-bit analog-to-digital converter.

### Procedure

Upon arrival to the laboratory and after providing informed consent, participants were fitted with the 256-channel Hydrocel Geodesic Sensor Net (EGI, Eugene, OR) and seated 65 cm in front of the computer monitor. A chin rest was used to minimize head movements and to maximize consistency of gaze distance and alignment to the monitor. After task instructions were provided to participants and once they understood the task, they performed a 20-trial practice block. Stimuli used in the practice block were not repeated during the actual study. The entire session, including recording set up, practice and experimental trials, lasted approximately 60 minutes.

### Event-Related Potential Processing

For derivation of the event-related potential (ERP), continuous EEG data were digitally filtered with a 30-Hz low-pass finite impulse response filter and then segmented relative to image onset (200 ms before and 800 ms after) and sorted according to image type (fragmented, scrambled) and perception (coherence, no coherence), as indicated by participants' response. A segment of the EEG was excluded from signal averaging if it was contaminated by ocular artifacts (e.g., 100 µV difference between EEG channels above and below the eyes for blinks and 100 µV difference between EEG channels near the outer canthi for lateral eye movements). Segments were also excluded if they contained 10 or more channels of data that exceeded a voltage threshold of 200 µV (absolute) or a transition threshold of 100 µV (sample to sample). After averaging, the data were re-referenced to the average reference. The number of trials that went into derivation of the average ERP waveforms for correct coherent perception and correct no coherent perception are 76 (SD = 21) and 33 (SD = 9), respectively.

### Joint Time-Frequency Analysis of Event-Related Potentials

We examined time-varying spectral changes with Morlet wavelets. In order to obtain good time and frequency resolution, two different constants were used to define the wavelet families: between 1–20 Hz (.5 Hz step), m = ƒ_0_/σ_f_ =  4, and between 21–80 Hz (.5 Hz step), m = ƒ_0_/σ_f_ =  7. Analyses were performed on the *unfiltered* ERP waveform (i.e., the raw EEG data were not filtered prior to segmentation and averaging) for each participant. The activity reflects time- and phase-locked (relative to the onset of the image) oscillatory activity (i.e., evoked oscillations). Time-varying energy in a given frequency band after stimulus onset was defined by Z-scores relative to the 200 ms pre-stimulus baseline.

### Source Estimates

Source estimates, describing neural sources of measured scalp potentials, were estimated with GeoSource (version 1.0) electrical source imaging software (EGI, Eugene, OR). See Luu et al. (2009) for specifics of GeoSource. For the ERP analyses, a single current density value for each source voxel (i.e., dipole) was computed as the root mean square (RMS) value over the 3 orthogonal (x, y, z) dipole moments for that voxel and averaged over the region of interest (ROI, see below). For the joint time-frequency (JTF) analyses, the JTF distributions were computed separately for each moment, such that the moment of each JTF result must be considered in the interpretation.

## Results

### Behavioral Data

A repeated-measures ANOVA model with Image Type (Fragmented, Scrambled) and Perception (Coherence, No Coherence) as within-subject factors was employed to analyze participants' median reaction time (RT) and endorsement rate. The analysis revealed a significant interaction for RT, F(1,21) = 16.01, p<.001. This interaction effect showed that when participants were presented with a fragmented image, their judgment about it containing a possible image (i.e., coherence) was faster (mean  = 778, SD = 164) than their judgment about it not containing a possible object (mean  = 903, SD = 210), t(21) = 5.36, p<.001. In contrast, when participants were presented with a scrambled image, they were faster at indicating that it does not contain a possible object (mean  = 836, SD = 177) compared to when they indicated that it does contain a possible object (mean  = 947, SD = 323), t(21) = −2.6, p<.02.

The analysis also revealed a significant interaction for endorsement rate, F(1,21) = 195.4, p<.001. Participants indicated that approximately 65 percent of the fragmented images contained a possible object (hits) whereas they judged the scrambled images to contain a possible object only 14 percent of the time (false alarms). From Signal Detection Theory, we can perform sensitivity and response bias analyses to assess participants' strategy. Sensitivity analysis, as measured by d' ( = 1.5), revealed that the separation between the mean of the signal (fragmented images) and noise (scrambled images) distributions is such that the task was not too difficult. Response bias, as measured by® β ( = 1.68) and C ( = .40), indicates that participants took a conservative approach (i.e., they set a high-threshold for indicating an image contained a possible object).

### ERP Data

**Figure 2:**
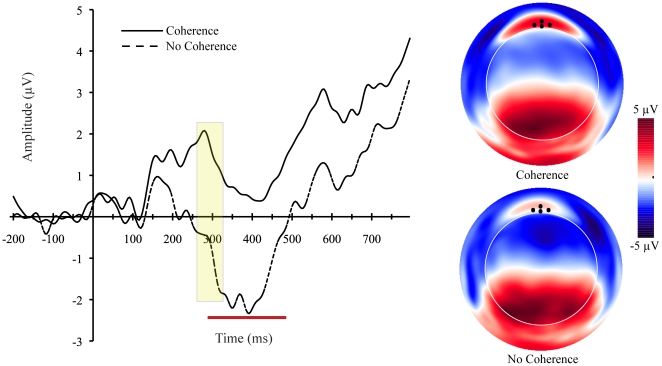
ERP waveforms and topographic maps. Left: Grand-average ERP waveform averaged across four frontal recording sites (indicated by black dots on the topographic maps on right). A “frontopolar P2” component is highlighted with yellow box and inversion of the P3 component is demarcated by red line. Right: Topographic maps at peak of P2. Black dots represent location of channels used to form the average waveforms. View of map is top looking down with nose towards top of image. Coherence  =  rated coherence for fragmented images; No Coherence  =  rated no coherence for scrambled images.

**Figure 3:**
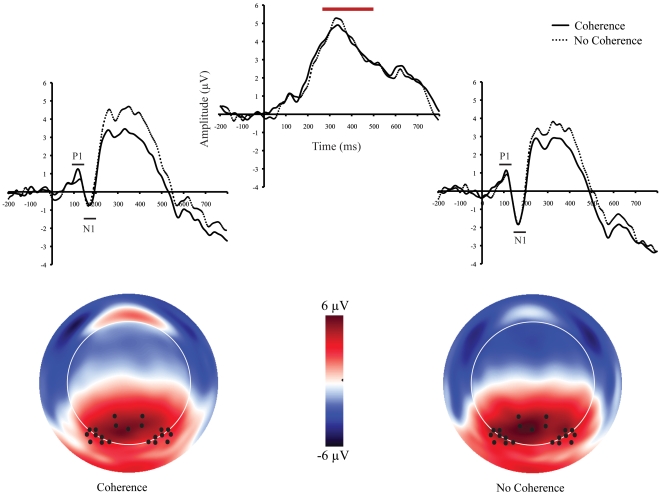
ERP waveforms and topographic maps. Top: Grand-average ERP waveform averaged across channel groups (indicated by black dots on the topographic maps on bottom). P1 and N1 components are demarcated by black lines and P3 component is demarcated by red line. Bottom: Topographic maps at the peak of the P3. Black dots represent location of channels used to form the average waveforms. View of map is top looking down with nose towards top of image. Coherence  =  rated coherence for fragmented images; No Coherence  =  rated no coherence for scrambled images.

Over frontal recordings sites at approximately 150 ms after image onset, the ERP associated with correctly perceived coherent images start to diverge from the ERP associated with scrambled images that were perceived to be non-coherent (Figure 2). At posterior regions, the ERPs associated with these two conditions began to diverge after the N1 component (∼200 ms, Figure 3). This divergence is seen as an attenuation of the P3 at bilateral occipital-temporal-parietal sites.

**Figure 4:**
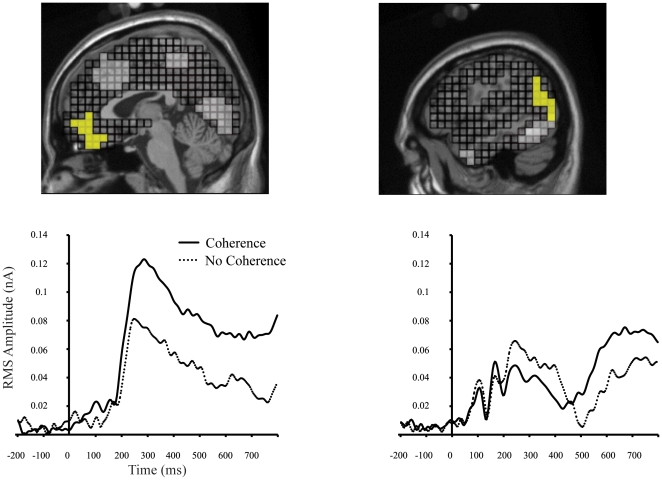
Source locations and source waveforms. Top: Location of OFC and Occipital-Temporal-Parietal ROIs (in yellow). Other ROIs can be seen in these two views (Left: ACC, PCC/Precuneus, and Lingual Gyrus; Right: Inferior Temporal Lobe, and Anterior Temporal Lobe). Bottom: source waveforms of grand-average activity for mOFC (left) and right TPO ROIs.

To analyze the ERP data, a *grand-average* difference waveform was generated by subtracting the ERP associated with correct coherent perception from the ERP associated with correct no coherent perception. Source estimation of the difference waveform was performed, and ROIs were defined based on the source solution (see Figure 4 and Table 1).

**Table 1:** Paired sample t Tests results for each region of interest across time.

ROI (Brodmann Area)	*X*	*Y*	*Z*	T1	T2	T3	T4	T5	T6	T7	T8	T9
Medial Orbitofrontal Cortex (mOFC)	−3	45	−13					<.009*	<.007*	<.03*		
R. Temporal-Parietal-Occipital	53	−67	15		<.02		<.02	<.004	<.004	<.002	<.007	<.07
L. Temporal-Parietal-Occipital	−52	−67	15									
R. Inferior Temporal Gyrus	53	−67	−6		<.06		<.04					
L. Inferior Temporal Gyrus	−52	−53	−13			<.1	<.05					
Posterior Cingulate Cortex/Precuneus	−3	−39	50		<.03	<.05						
R. Inferior Parietal	32	53	50									
L. Inferior Parietal	−38	−53	50									
R. Anterior Temporal Lobe	39	−4	−34									
L. Anterior Temporal Lobe	−38	10	−34									
Lingual Gyrus	4	−74	8		<.008	<.09	<.004					
Anterior Cingulate Cortex (ACC)	−3	24	43		<.002	<.04						
R. Inferior frontal Gyrus/Insula	39	17	−13									
L. Inferior frontal Gyrus/Insula	−38	17	−13									

T1: 0–100 ms; T2:50–150 ms; T3:100–200 ms; T4: 150–250 ms; T5: 200–300 ms; T6: 250–350 ms; T7: 300–400 ms; T8: 350–450 ms; T9: 400–500 ms.

*Significance levels reported as one-tailed tests.

Each participant's ERP data were submitted to GeoSource, and source activity was estimated separately for coherent and non-coherent conditions. To compare the time course of difference between the two conditions for each ROI, an average RMS over a 100 ms-wide window was computed. This was done for the time interval between time 0 and 500 ms after image onset, with a 50% overlap for each window (e.g., T1 = 0–100 ms, T2 = 50–150, etc.). Paired-sample t-tests were employed. Because we began with the specific hypothesis that perception of coherence would be differentiated within the mOFC (stronger activation for coherent perception), the significance levels are reported as a one-tail test for this ROI; for all other ROIs significance levels are evaluated and reported as two-tail tests. In order to minimize false-positive error rates, we only consider ROIs that exhibit a statistically significant difference in two contiguous time windows. Results of the ROI analysis are presented in Table 1.

We performed correlation analyses to investigate the relation between the activity in the mOFC and right temporal-parietal-occipital (TPO). We did this separately for the coherent and non-coherent conditions. For the coherent condition, the pattern of correlations shows that early TPO activity (T2 and T3) was correlated with mOFC activity at T5. At T4 and T5 TPO activity did not significantly correlate with mOFC activity at anytime. Beginning at T5, mOFC activity correlated with TPO activity at T6-T8 (see Table 2). These correlation patterns are remarkable in that they suggest a pattern of influence from the TPO to the mOFC and from the mOFC back to the TPO.

**Table 2:** Correlations between TPO and mOFC across time.

	T5 mOFC	T6 mOFC	T7 mOFC
T2 R TPO	**r = .46, p<.032**		
T3 R TPO	**r = .43, p<.05**		
T4 R TPO	r = .35, ns	r = .27, ns	r = .18, ns
T5 R TPO	r = .40, ns	r = .33, ns	r = .23, ns
T6 R TPO	**r = .48, p<.024**	**r = .42, p = .05**	r = .30, ns
T7 R TPO	**r = .45, p<.038**	**r = .41, p<.06**	r = .30, ns
T8 R TPO	**r = .42, p = .05**	r = .37, ns	r = .30, ns

T1: 0–100 ms; T2:50–150 ms; T3:100–200 ms; T4: 150–250 ms; T5: 200–300 ms; T6: 250–350 ms; T7: 300–400 ms; T8: 350–450 ms; T9: 400–500 ms.

No significant correlations between the mOFC and TPO were observed at any time point in the non-coherent condition. Furthermore, to verify the specificity of this pattern of correlation between mOFC and TPO source activity, we examined the relation between the mOFC and other ROIs. Activity at mOFC was not correlated with activity from any other ROI at any time point, with the one exception of activity from the left inferior temporal gyrus at T3 with the OFC at T5 (r = .43, p<.05).

### Evoked Spectral Changes

**Figure 5:**
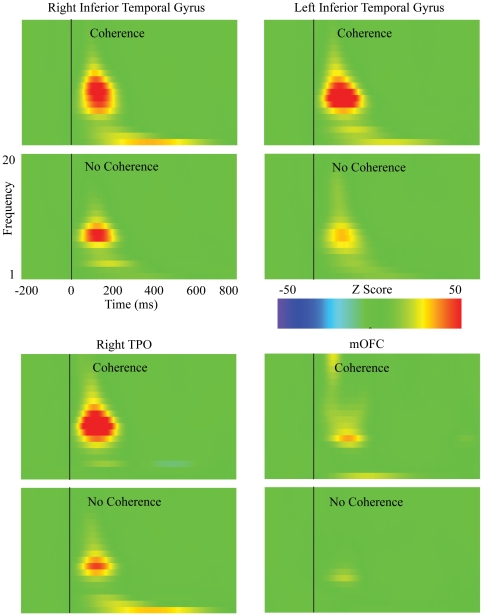
Time-Frequency plots of evoked oscillatory activity between 1–20 Hz. Energy increases and decreases after stimulus onset (time 0) are defined as standard deviations from the baseline period (−200–0 ms).

Several sources demonstrated increased energy in the alpha-band (8–12 Hz) approximately 50 ms after the onset of a fragmented image rated as coherent compared to a scrambled image rated as not coherent, and the energy increase lasted for approximately 200 ms (see Figure 5). In particular, these sources show strong energy increases in the alpha frequency for the anterior-posterior vector, which reflect the orientation of the ROI that accounts for the P1-N1 (for left and right inferior temporal gyrus and TPO) and P2 (for mOFC).

**Figure 6:**
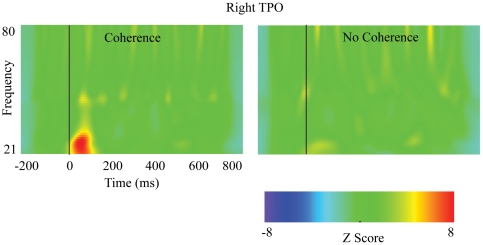
Time-Frequency plots of evoked oscillatory activity between 21–80 Hz. Energy increases and decreases after stimulus onset (time 0) are defined as standard deviations from the baseline period (−200–0 ms).

Between 21 and 80 Hz (i.e., gamma-band) there were several ROIs that also demonstrated energy increases after stimulus onset in response to an image rated as coherent. Most notable is the energy increase observed for the right TPO approximately 50 ms after image onset and lasting for approximately 100 ms (see Figure 6). Examining the source vectors in the x, y, z orientations, the anterior-posterior vector orientation of this ROI demonstrated the largest energy increase.

To determine whether observed energy increases in the alpha and gamma bands in response to coherent perception are statistically reliable across participants, we obtained, in each subject, average Z-score between 50–200 ms after stimulus onset across 8–12 Hz (alpha-band activity) and 21–40 Hz (for gamma-band activity) for each ROI. We then performed paired sample t-tests on each ROI.

In the alpha-band, only left inferior temporal and right TPO ROIs exhibited a significant energy increase in response to fragmented images rated as coherent, t(21) = 2.2, p<.04 and t(21) = 2.3, p<.04. There was a trend for greater activity in the right inferior temporal gyrus in coherent versus non-coherent judgments, t(21) = 1.9, p<.07. Given that peak difference in mOFC activity between perception of coherence and non-coherence occurred at approximately 250 ms after image onset (see Figure 4 and Table 2), we performed a paired comparison t-test of this ROI for the window spanning 250–350 ms (T6) after image onset. This analysis revealed a significant increase of alpha in this interval for coherent judgments, t(21) = 2.4, p<.03. For gamma-band activity, only the right TPO demonstrated a significant increase for coherent judgments, t(21) = 3.6, p<.003.

## Discussion

### Behavioral Evidence of Processing Strategy

In this paradigm, we, like Volz and von Cramon [8], emphasized to participants that they did not need to be able to identify the object within an image. Rather, we encouraged them to use their feeling (i.e., impression) of whether or not an image contained a possible object. Given this instruction to guess at the presence of a possible object, participants were still relatively accurate, reporting that 65% of fragmented images contained a possible object compared to 14% for scrambled images. Behaviorally, RTs associated with hits and correct rejections were much faster than those associated with misses and false alarms, respectively. Measures of sensitivity and response bias revealed that participants took a conservative strategy that minimized false alarms, perhaps due to the fact that it was relatively easy to discriminate between fragmented and scrambled images.

These behavioral results suggested that our participants were better able to guess correctly when they were not certain than those participants examined by Volz and von Cramon [8]. Volz and von Cramon found that participants in their study were much *slower* when indicating that a fragmented image contained a possible object, and their participants only endorsed 33.3% of fragmented images as containing possible objects. Volz and von Cramon interpreted their behavioral findings to indicate that their participants did not adopt a low-response threshold, but rather employed a strategy emphasizing correct rejections. Our participants also employed a strategy that emphasized correct rejections (86% compared to 84% from Volz and von Cramon), but they also seem to be more intuitive in their decisions, as reflected in the larger percentage of hits (65% compared to 33.3%) and faster RTs. Thus, in this task, behaviorally, intuition may be reflected in faster RTs and more accurate performance for fragmented images rather than adoption of a low-response threshold.

### Reentrant Constraints on Visual Perception

Despite the behavioral differences, we confirmed the findings by Volz and von Cramon that the mOFC is involved in the initial perception of coherence. With the time course resolved by dEEG, we could determine that activity in the mOFC began to differentiate between coherent and non-coherent percepts at approximately 200 ms, around the time that a positive frontopolar peak appeared in the head surface topography. In visual perception tasks, great care must be taken to rule out ocular artifacts that may contaminate the ERP, particularly for those ERP components distributed over the frontal recording sites. With regards to the present study, saccade-related artifacts are potential concerns. The short duration (400 ms) of stimulus presentation helps to minimize eye movements. Although it is true that participants can make saccades prior to the offset of the images (average saccades tend to occur between 200–300 ms after stimulus onset), this is only an issue in ERP studies if saccades are of large amplitudes or strictly time-locked to the stimulus. If they are of small amplitudes and are not time-locked to stimulus onset, they are cancelled in the averaging process. In fact, Yuval-Greenberg and colleagues [31] extensively studied this issue and noted that saccades are usually problematic for studies that analyze induced (i.e., single-trial) EEG activity. Moreover, because saccades have characteristic topographic distributions, they are not easily mistaken as ERPs in the averaged data. That is, saccades are characterized by voltage deviations (in opposite directions) in channels near the external canthi. This pattern is not observed in the average data (see Figure 2).

Volz and von Cramon [8] interpreted their mOFC finding in relation to Bar's [3] top-down model of visual object recognition. In this model, a partially processed, low spatial frequency version of an image is communicated to the mOFC via the dorsal cortical pathway. The information activates networks within the mOFC, providing possible memory-guided interpretations of the image. These mOFC patterns are back-projected to the inferior temporal cortex to constrain further processing of the image, rapidly facilitating the process of object recognition. Using magnetoencepalography (MEG) to examine brain activity during an object *recognition* task, Bar et al., [9] observed activity within the mOFC at approximately 130 ms after image onset when participants recognized an object. mOFC activity preceded activity in the right and left inferior temporal cortices, regions known to be involved in object recognition, by about 50 and 851 ms, respectively. Consistent with the model, these researchers also found strong phase synchrony (in the 8–12 Hz band) at 80 ms and 130 ms after stimulus onset between mOFC and inferior temporal cortex, suggesting that these two regions directly interact at these two time periods.

In the present study, for images that were appraised as containing possible coherent objects, we found that activity from the right TPO, starting at 50 ms after image onset, predicted activity in the mOFC at about 200 ms after stimulus onset, which is the time that activity in the mOFC begins to discriminate between coherent and non-coherent perception. The implication is that early processing in visual cortex leads to the appraisal of coherence mediated in part by the mOFC. Once activity in the mOFC region was engaged by appraisal of coherence, starting at about 200 ms, the mOFC activity at this time then predicted activity that would occur in the TPO at about 250 ms after stimulus onset. The predictive mOFC-TPO relation then lasted for approximately 250 ms.

It may be surprising that it was the TPO rather than inferior temporal visual cortex that predicted the mOFC response. Given that this task involved object perception, a function of the ventral occipital-temporal pathway, why was the initial predictive response seen in TPO, a region that is unique in combining inputs from both ventral (object) and dorsal (configural) visual pathways? The answer may be that both object and configural processing are required for perceptual operations involved in discerning object patterns within the fragmented line drawings of the present experiment. The right hemisphere has been suggested to be preferentially involved in the processing of low spatial frequency information [18], and recent findings show that configural relations are embedded in low spatial frequency information [19]. The TPO has been proposed to be involved in the coding of spatial relationships [20] as well as in allocentric visuospatial attention [21]. Therefore, the need for representing low spatial frequency information, combined with the need for configural integration, likely led to engagement of the right TPO region in the present experiment.

Volz and von Cramon [8] also reported that activity in the ventral-temporal-occipital (VTO) regions differentiated between coherent and non-coherent perception, but they did not find functional correlation between the VTO and mOFC. Similarly, we also found that activity in the left inferior temporal lobe differentiated coherent from non-coherent perception (see Table 2 and joint-time-frequency results) and that activity in this region does not correlate with mOFC activation. It is noted, however, that the inferior temporal region identified in the present study is not the same region identified by Volz and von Cramon as VTO. Thus, in the present task low-spatial frequency configural information appears to contribute directly to initial perception of coherence via its influence on mOFC activity whereas activity from the inferior temporal lobe does not. Volz and von Cramon proposed that the VTO is involved in the actual perception of the object and not just the experience of the physical stimulus. If this is true, we would expect the time course of VTO activity to lag behind mOFC activation.

We also found that judgment of coherence was associated with early activity in lingual gyrus, PCC, and the ACC. The lingual gyrus and PCC appear to contribute to object processing in the fusiform gyrus, facilitating the transfer of contour and shape information to other areas involved in object recognition [20]. Considering the early activity in the ACC that was greater for non-coherent stimuli, we have proposed that the ACC is involved in contextual representation of task requirements, with these representations forming expectations that guide performance [22]. In this light, it may be that greater ACC activity to the non-coherent stimuli reflected the greater effort to resolve the appraisal of non-coherence, compared to the faster and presumably less effortful appraisal of coherence.

### Oscillatory Dynamics

We found that evoked (phase-locked) alpha-band activity increased significantly for appraisals of coherence, in contrast to appraisals of non-coherence. This increase occurred at the time of the P1-N1 of the ERP, although P1-N1 amplitudes did not differ between these two conditions. This oscillatory effect may be consistent with the proposal that the N1 reflects phase-reset of the ongoing alpha rhythm to the onset of a stimulus [23]. Interactions between distant cortical areas in electrophysiological oscillations, particularly in the theta and alpha bands, have been interpreted to reflect top-down processes in visual perception [24], [9].

Evoked gamma band responses (eGBRs), which occur no later than150 ms after stimulus onset [25], are thought to reflect local neuronal activity and sensory binding [24]. These more bottom-up processes may be modulated by top-down influences [25]. eGBRs have been shown to be sensitive to stimulus features, such as size and eccentricity [26], and they can be observed in primary as well as secondary visual areas (such as V5) [27]. Hermann and colleagues noted that eGBRs are mainly observed in regions where the computation occurs. The significant and relatively focal eGBR in the TPO region in the present experiment is consistent with the interpretation that the TPO achieves primary binding of the configuration of fragmented lines.

### Intuition as the Initial Perception of Coherence

Volz and colleagues [8], [28] have argued that the initial perception of object coherence, as reflected by activity in the mOFC, is functionally equivalent to intuition. Coherence in this sense is similar to a pattern, meaning, or structure that exists within an information stream, and when it is not consciously represented can be taken as reflecting the process of intuition [2]. It is interesting that the mOFC appears to be an important region of frontolimbic cortex that participates in the subjective appraisal of perceptual coherence. Interconnectivity of the mOFC with the insula, amygdala, thalamus, and hypothalamus link it closely with the representation of viscerosensory experiences [29], [6]. Thus, it is not surprising that the mOFC has been shown to contribute to many forms of behavioral learning, particularly those requiring visceral, hedonic, or affective discriminations [6], [30]. This line of evidence, coupled with the findings that appraisal of coherence occurs before the act of object identification (because this appraisal is activated via fast pathways that initially bypass bottom-up analysis), explains why the feeling of knowing is often associated with an affective experience: the feeling of knowing arises at the gut level.
